# Supplementary material for: Growth Mechanism of Periodic-Structured MoS2 by Transmission Electron Microscopy
Source: Nanomaterials (Basel). 2021 Dec 31;12(1):135. doi: 10.3390/nano12010135 (PMC8796029; doi:10.3390/nano12010135)
Supplement: Supplementary file 1 [file nanomaterials-12-00135-s002.zip › SI_nanomaterials-1492825-1.pdf]

nanomaterials

Article

Growth Mechanism of Periodic Structure MoS_2 by Transmission Electron Microscopy: Supplement Material

Arvind Mukundan[1], Yu-Ming Tsao [1], Sofya B. Artemkina [2,3], Vladimir E. Edorov [2,3] and Hsiang-Chen Wang [1,*]

[1] Department of Mechanical Engineering, Advanced Institute of Manufacturing with High tech Innovations (AIM-HI), and Center for Innovative Research on Aging Society (CIRAS), National Chung Cheng University, 168, University Rd., Min Hsiung, Chia Yi 62102, Taiwan; d09420003@ccu.edu.tw (A.M); d09420002@ccu.edu.tw (Y.M.T.)

[2] Nikolaev Institute of Inorganic Chemistry, Siberian Branch of Russian Academy of Sciences, Novosibirsk 630090, Russia; artem@niic.nsc.ru (S.B.A.); fed@niic.nsc.ru (V.E.F.)

[3] Department of Natural Sciences, Novosibirsk State University, 1, Pirogova str., Novosibirsk 630090, Russia.

* Correspondence: hcwang@ccu.edu.tw

Citation: Mukundan, A.; Tsao, Y.-M.; Artemkina, S.B.; Fedorov, V.E.; Wang, H.-C. Growth Mechanism of Periodic-Structured MoS2 by Transmission Electron Microscopy. Nanomaterials 2022, 12, 135. https://doi.org/10.3390/nano12010135

Academic Editor(s): Akinobu Yamaguchi

Received: 18 November 2021
Accepted: 30 December 2021
Published: 31 December 2021

Publisher's Note: MDPI stays neutral with regard to jurisdictional claims in published maps and institutional affiliations.

Copyright: © 2022 by the authors. Licensee MDPI, Basel, Switzerland. This article is an open access article distributed under the terms and conditions of the Creative Commons Attribution (CC BY) license (https:// creativecommons.org/licenses/by/ 4.0/)

1. Obtaining the layers of MoS_2

The most common research methods used to obtain the layers of MO_2 are Mechan-ical Exfoliation Method, Chemical Vapor Deposition, Electrochemical Lithiation Process and Sulfurization of MoO_3. Among them, the chemical vapor deposition method can efficiently grow a large area and uniform single-layer MoS_2 film, which is also the most commonly used process method is employed in this current research.

1.1. Chemical vapor deposition (CVD)

The use of CVD can control high-quality MoS_2 morphology, number of layers and uniform and large-area thin films. For the appearance of MoS_2, the shape of the area on the substrate will evolve with the ratio of Mo to S (1:>2, 1:2, and 1:<2), and through SEM, Raman spectra, AFM and PL. The test results confirmed that the CVD method can grow a uniform and highly crystalline single-layer MoS_2 film.

2. Instrument Specifications

2.1. Micro-Raman Spectroscopy

Raman spectra is a method that can effectively analyze the molecular structure, molecular vibration mode, and rotation mode to study the determination of the crystal lattice and the position of molecules or chemical bonds. Raman scattering uses the interaction of excitation light and matter to produce inelastic scattering. The general laser range is visible light, near-infrared light, or near-ultraviolet light. In order to understand the number of layers of MoS_2 film, Raman spectrometer is used. The model of the Raman microscope used in this experiment is HORIBA XploRA ONE. The Raman microspectrometer uses a 532nm laser to enter the sample. The photons in the laser will collide with the molecules in the sample material to generate Raman scattered light, which is received by the instrument After measuring the molecular vibration. For example, the MoS_2 film, the E_{12g} and A_{1g} vibration modes are the main signals for the judgment of MoS_2. The two vibration modes are highly dependent on the thickness of MoS_2. When the k value of the subtraction of E_{12g} and A_{1g} peaks is less than $20cm^{-1}$, the analyzed MoS_2 is regarded as a single-layer structure.

2.2. Micro-Raman Micro-PL spectrometer

Micro-Raman and low-light excitation fluorescence spectrometers are Horiba Jobin Yvon LabRAM HR systems. It has a powerful non-destructive spectrum measurement function, which can separately analyze the light-excited fluorescence spectrum and

Raman spectrum. The light source is 633nm, 532nm and 325nm lasers. The Raman spectrum ranges from 150 to 5000cm^{-1}. It is capable of two-dimensional image scanning, low temperature (77 - 300 K), polarization measurement, and the sample can be measured without additional complicated pre-processing. From the measurement of light-excited fluorescence spectra, important information such as transmission path, energy gap size, band structure, doping impurity types, carrier transition behavior, composition of compounds, size effects and carriers in nanomaterials can be obtained. The measurement of Raman spectroscopy can be applied to different fields. Many occasions that require non-destructive, microscopic, chemical analysis and imaging analysis are involved. Raman can be used to quickly detect its chemical composition and structure. In addition to being used as a basis for judging the structure, composition, and quality of materials, it is also often used in the fields of carbon materials, semiconductor materials, pharmaceuticals and cosmetics, life sciences, geology, and mineralogy. The PL measurement results in this study uses a 532nm laser as the excitation light source to measure the PL of a single-layer MoS_2 film. The measurement results show that a strong signal is generated at the wavelength of 667nm, and the energy is 1.84eV after conversion. It is the energy gap of single-layer MoS_2.

2.3. Optical Microscope

Optical microscope (OM) uses an optical lens to produce image magnification effect. The light incident from the object is magnified by the objective lens and eyepiece of the optical system. There are 5X, 10X, 40X and 100X on the objective lens plate while the magnification can be changed as required. According to the design of condenser and objective, optical microscopes can be divided into reflective and transmissive types according to different samples. Reflective microscopes are generally used for opaque samples. After the light is irradiated on the object, the light reflected by the object enters the microscope to obtain an image, which is mostly used to observe solids, materials, etc. The transmission microscope is for the transparent or very thin state of the sample itself, so that the light can enter the microscope from the sample. The image is generally used to observe biological tissues. The stage is the platform that carries the sample. There is an adjustable aperture below it. When the light is dark, you can adjust the aperture to choose a larger aperture.

2.4. Atomic Force Microscope (AFM)

Atomic force microscope (AFM) is a nano-level high-resolution scanning probe technology. Through observation atoms and molecules can be visually seen. The micro cantilever is used to sense and amplify the Van der Waals force between the tip of the cantilever and the atoms of the sample under test to further show the surface characteristics of the sample. When the atom is very close to each other, the repulsive force of the electron cloud is greater than the attractive force between the nucleus and the electron cloud, making the entire net force behave as a repulsive force. On thecontrary, if two atoms are separated by a certain distance, the repulsive force of the electron cloud between the two will be less than the attraction between the nucleus and the electron cloud of each other. The application of AFM technology is quite simple, no additional processing (such as platinum plating, baking) is required for the sample, and the damage to the sample is less than other detection technologies, and it can also be measured in a variety of environments (such as air, liquid). Live cells can also be dynamically observed, but unlike electron microscopes, AFM can provide three-dimensional images, and AFM imaging has a smaller range and slower speed than SEM. AFM measurement can be divided into three types of operations based on the distance between the probe and the sample: contact, non-contact, and percussion. The measurement system is a non-contact measurement when the distance between the tip of the atom and the atom on the sample surface.

2.5. Dual-beam focused ion beam

The dual-beam focused ion beam microscope, model FEI Nova-200 NanoLab Compatible, is used in this study. The magnification is about 1500 times that of the optical microscope. The magnification can reach more than 10,000 times and the depth of field is large. It is mainly used to observe the microstructure of the sample surface and section. Focused Ion Beam (FIB) uses a well-focused ion beam for the sample to be observed for modification and image acquisition. FIB is mainly used to obtain very accurate sample cross-sections or perform circuit modification after imaging through SEM, STEM and TEM. In addition, FIB can detect emitted electrons from ion beams or electron beams for direct imaging. The contrast mechanism of FIB is different from SEM and STEM, so in some cases unique structural information can be obtained. Dual Beam combines the FIB/SEM two technologies into one tool, using FIB to prepare samples and using SEM, TEM or STEM instruments to obtain electronic images, while the FIB of single beam has only one ion beam source. The principle of scanning electron microscope is to apply an external electric field and accelerating voltage to make the electron gun generate a high-energy electron beam through the principle of thermal dissociation or field emision. It is focused on the sample through an electromagnetic lens, and the deflected electron beam is made two degrees on the surface of the sample by the scanning coil. In space scanning, when the electron beam interacts with the sample, it will emit various signals, such as secondary electrons, backscattered electrons, absorbed electrons, etc. Generally, the electron beam is focused on the sample, and the electrons on the sample surface will produce elastic collisions and non-uniformity. Elastic collision, and the information with the surface topography is received and imaged by the secondary electron detector, so the image of the unevenness of the sample surface is obtained.

2.6. Tube furnaces

The high-temperature furnace tube used in this research has three heating zones. The working principle is that the material generates steam through heating, and the high-quality large-area thin film is grown by one or more precursors chemically reacting on the surface of the substrate. The most common and efficient way to generate large-area MoS_2 film, the three-zone furnace tube machine model we use is Linberg/Blue HTF55347C. The heating zone temperature can reach up to 1200C, and the heating rate Up to 20C/min, argon and nitrogen can be introduced, and up to eight different temperature parameter configurations can be set according to the experiment.

2.7. Transmission Electron Microscope (TEM)

The high-resolution transmission electron microscope (TEM) model is PHILIPS CM-200 TWIN which can observe the morphology of the material and the fine appearance, lattice structure and defects inside the material. The electron beam operating at 20-200KV has a wavelength of 0.0251nm and a point resolution of about 0.27nm, which is suitable for observing atomic distance images. By using a high-energy electron beam (100KeV-1MeV) to penetrate the material to be tested, and the internal structure of the material will produce elastic scattered electrons and inelastic scattered electrons. The size of the scattering angle is related to the density and thickness of the object, because the electrons can be collected and elastically scattered to obtain the information of the atomic arrangement; the information of the material composition and bonding can be obtained by inelastic scattering, and then the contrast of light and dark can be obtained through the combination of different optical lenses. After obtaining the contrast of light and dark images through different optical lens combinations, they are displayed by a CCD imaging system. In addition, an X-ray energy spectrum dispersion analyzer (EDS) and an electron energy loss spectrometer (EELS) can be added to detect the difference in the material.

3. Transmission Electron Microscope Analysis

Since the depth of the periodic holes in the substrate is set to 300 nm, to understand the growth of MoS_2 to cover the surface of the substrate, a high-magnification image maps is required to analyze the profile. Therefore, the use of TEM observation is a necessity.

3.1. Substrate Profile Analysis

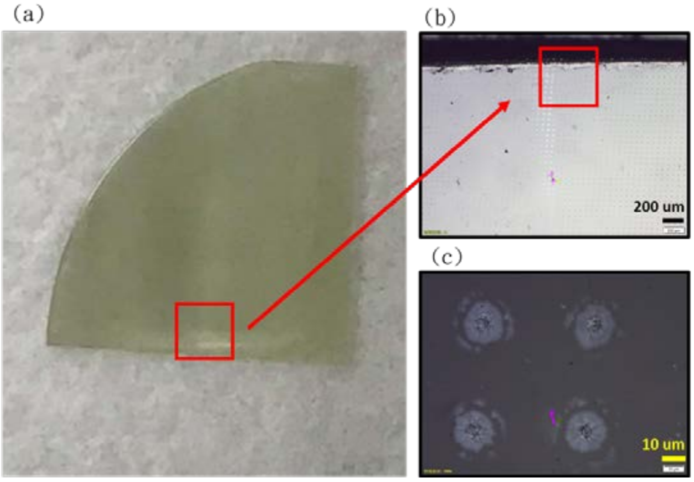

Figure S1. (a) An image of a sapphire substrate with periodically growing MoS_2, (b) is a partially enlarged image of Figure (a), and (c) is an enlarged OM image of the red box in Figure (b).

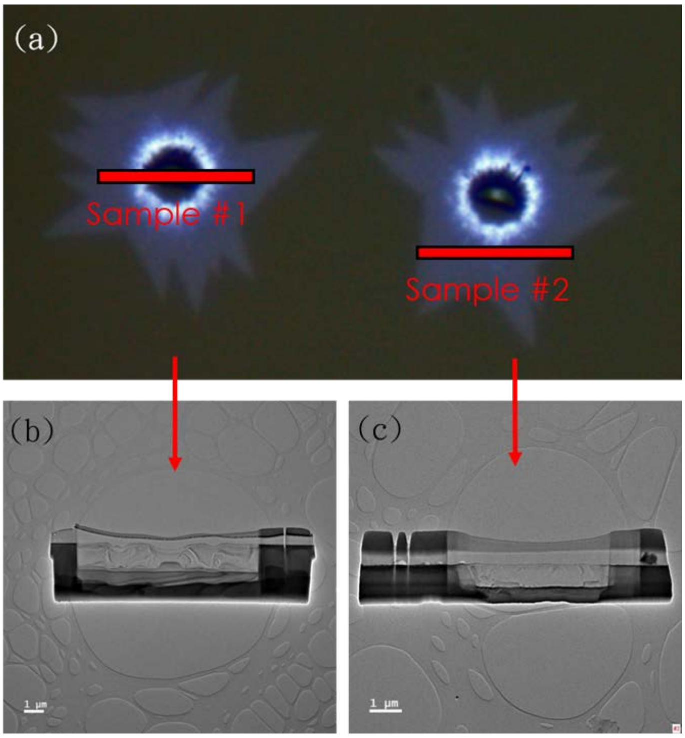

Figure S2. (a) OM image of periodically growing MoS_2, (b) is the SEM image of the cross section of picture (a) marked by the red line, which is defined as sample#1, (c) is the SEM image of the cross-section at sample#2 where the red line marks the sampling defined as sample#2 in Figure (a).

Figure S1 shows the sampling position for TEM measurement. Figure S1(a) is an image of a periodically grown MoS_2 sapphire substrate, and Figure S1(b) is a partially enlarged image of Figure S1(a). It can be seen that there are large-area periodic holes. Figure S1(c) is an enlarged OM image at the red box in Figure S1 (b). We can see that 4 holes exist, and there is some kind of film around the holes. Figure S2 is a more detailed

description of the sampling position of Figure S1 in TEM measurement. Figure S2(a) is an OM image of periodically growing MoS_2. The red line indicates the part using the focused ion beam to obtain a cross-sectional view of the sampling location. There are two main sampling locations, one crosses the hole, and the other does not cross the hole. Figure S2 (b) shows the red line in Figure S2 (a), which is defined as the cross-sectional SEM image of sample#1, Figure S2 (c) is the red line in Figure S2(a), which is defined as the cross-sectional SEM image of sample2.

Figure S3(a) is a cross-sectional SEM image of sample#1, and Figure S3(b) is a schematic diagram of the surface undulation curve of Figure S3(a). As the cross-cut holes are complicated, they are named for the convenience of interpretation. The red circled part is named hole, the purple circled area is named edge of hole, and the green circled irregular ring around the hole. The ring up to the nearly flat depression is named the edge of ring.

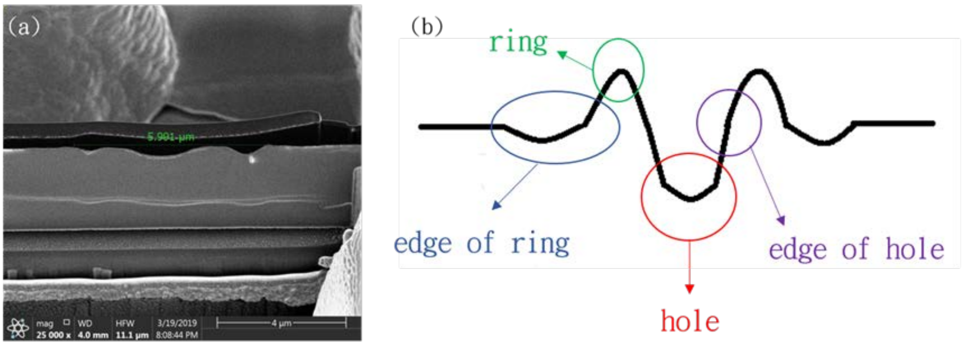

Figure S3. (a) A cross-sectional SEM image of sample#1, and (b) is a schematic diagram of the nomenclature of the section in Figure (a).

Figure S4(a) shows a schematic image of the cross-section of sample#1. The arrows of different colors will be used for HRTEM images. The purple arrow to the left represents the flow direction of argon. Figure S4(b) shows the HRTEM image marked with the red arrow in Figure S4(a). The position is the farthest from the hole. The yellow arrow points to the MoS_2 multi-layered growth. Figure S4(c) is the HRTEM image marked with the green arrow in Figure S4(a). The position is relatively close to the hole compared to Figure S4(b). The yellow arrow points to the position where you can see multiple layers close to the surface. The number of growth layers of MoS_2 is multi-layer growth, and slightly more. Figure S4(d) is the HRTEM image marked with the blue arrow in Figure S4(a). The position is the edge of the ring. The part indicated by the yellow arrow shows that not all MoS_2 grows on the surface. In the same direction, it can be seen that the growth of MoS_2 is formed by overlapping flakes.

Figure S5(a) is a schematic image of the cross-section of sample#1. The arrows of different colors will be used for HRTEM images. The purple arrow to the left represents the flow direction of argon. Figure S5(b) is the HRTEM image marked with the red arrow in Figure S5(a). It can be seen that the multi-layered MoS_2 grows near the surface, and the yellow arrow indicates the overlapping part of MoS_2 grown in different directions. Figure S5(c) is the HRTEM image marked with the green arrow in Figure S5(a). The position is in the ring. The yellow arrow indicates that the bottom layer is close to the sapphire substrate and is a non-single crystal layer. The following chapters will proceed discuss further than the comparison. Figure S5(d) is the HRTEM image marked by the blue arrow in Figure S5(a). It is the edge of hole. The yellow arrow points to the growth of MoS_2 with high levels in the middle, but the upper part is covered. Before the material analysis, the substance is not sure to be called the mixed zone.

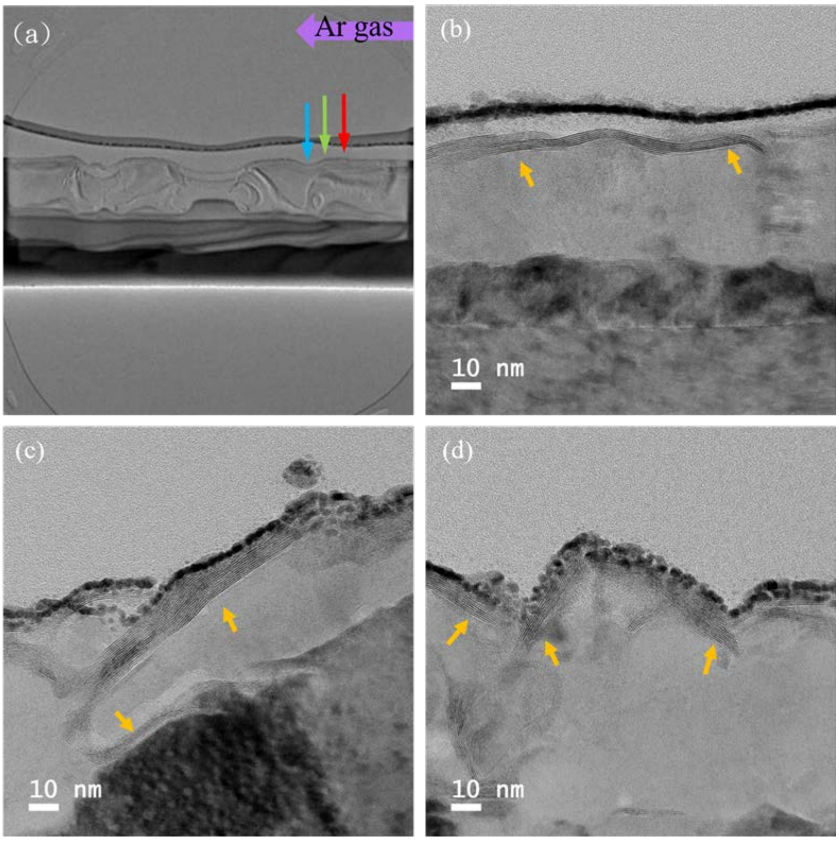

Figure S4. (a) is a schematic image of the cross section of sample#1, (b) indicates the HRTEM image at the red arrow in figure (a), (c) indicates the HRTEM image at the green arrow in figure (a), (d) indicates the HRTEM image at the blue arrow in figure (a).

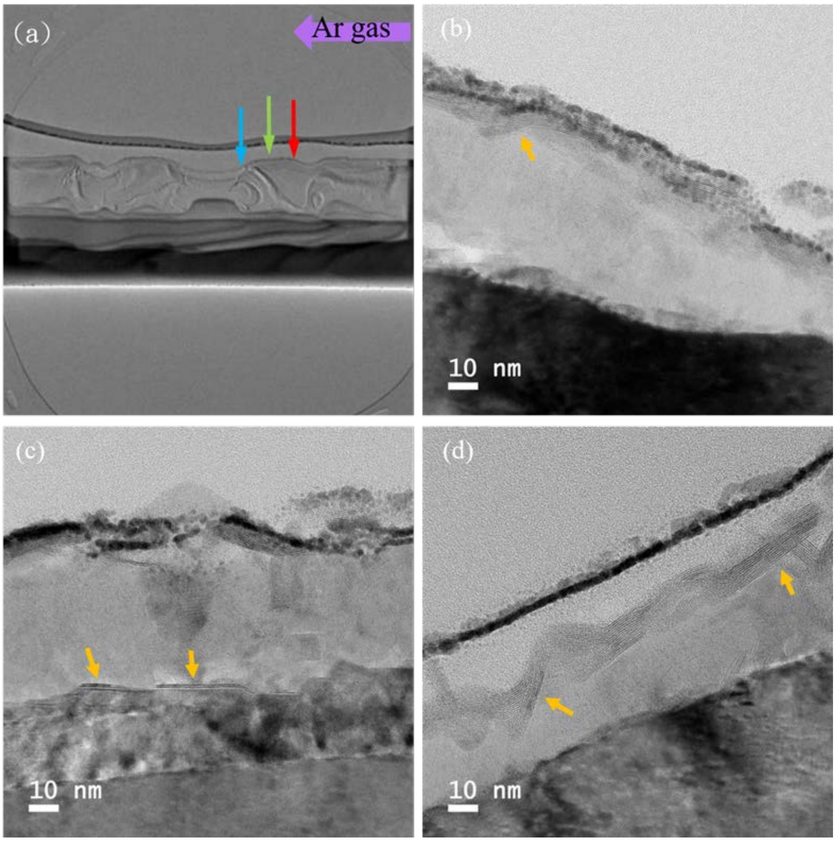

Figure S5. (a) is a schematic image of the cross section of sample#1, (b) indicates the HRTEM image at the red arrow in figure (a), (c) indicates the HRTEM image at the green arrow in figure (a), (d) indicates the HRTEM image at the blue arrow in figure (a).

Figure S6(a) is a schematic image of the cross section of sample#1. The arrows of different colors will be used for HRTEM images. The purple arrow to the left represents the flow direction of argon. Figure S6(b) is the HRTEM image marked with the red arrow in Figure S6(a). It is at the bottom of the hole, which is relatively flat, and the presence of MoS_2 is almost invisible. Figure S6(c) is the HRTEM image marked with the green arrow in Figure S6(a). At the edge of hole, the orange arrow points to the thicker MoS_2 at the raised corner, and the yellow arrow points to the thicker MoS_2. MoS_2 is grown thinner, so it can be found that multiple layers of MoS_2 will only accumulate at the edge of the hole, and the number of growth layers of MoS_2 at the steeper place is less. Figure S6(d) is the HRTEM image of marked by the blue arrow in Figure S6(a). The selected area is in the ring. The yellow arrow points to the area where the growth layer of MoS_2 is found to be quite thick and the flakes are crossed and stacked together.

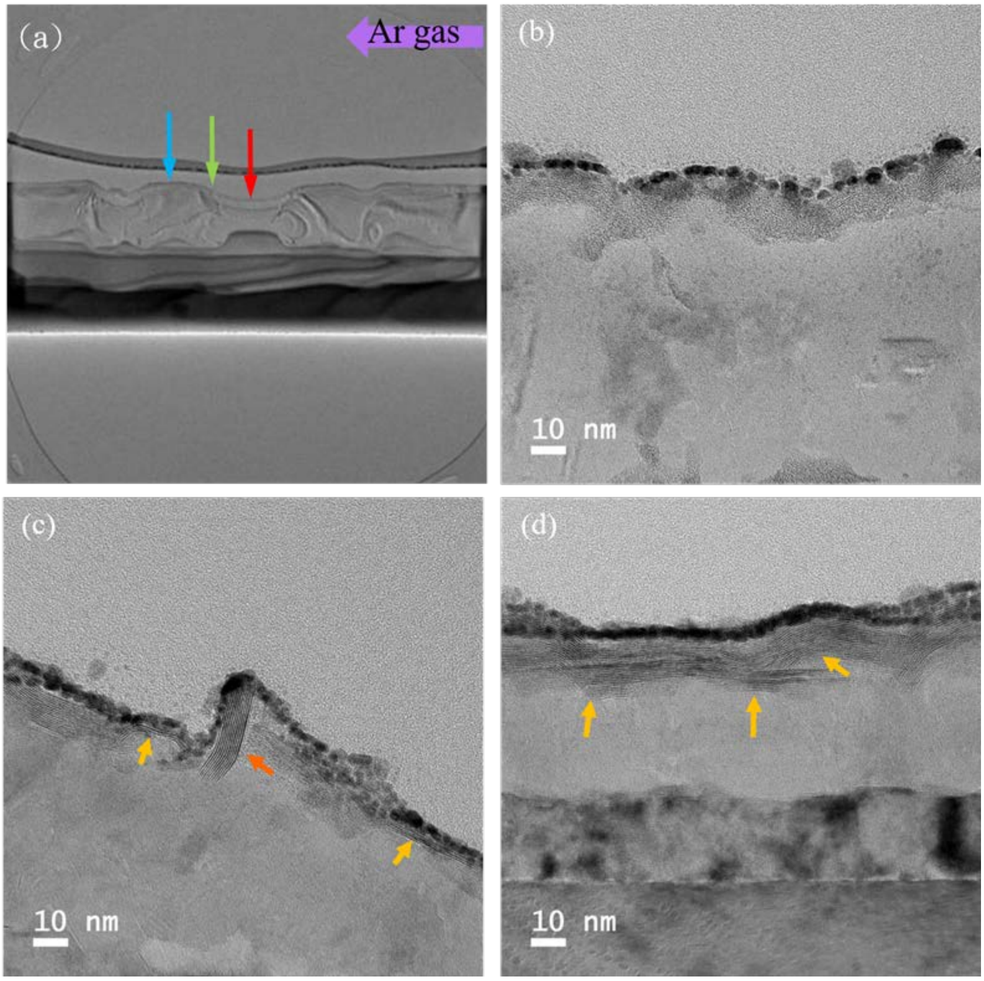

Figure S6. (a) is a schematic image of the cross section of sample#1, (b) indicates the HRTEM image at the red arrow in figure (a), (c) indicates the HRTEM image at the green arrow in figure (a), (d) indicates the HRTEM image at the blue arrow in figure (a).

Figure S7(a) is a schematic image of the cross-section of sample#1. The arrows of different colors will be used for HRTEM images. The purple arrow to the left represents the flow direction of argon. Figure S7(b) is the HRTEM image marked with the red arrow in Figure S7(a). The yellow arrow indicates the area where the multi-layered MoS_2 growth overlaps. Figure S7(c) is the HRTEM image of Figure S7(a) marked by the green arrow, and the yellow arrow marked by the multi-layered MoS_2 growth. Figure S7(d) is the HRTEM image of Figure S7(a) marked with the blue arrow, and the yellow arrow marks the position where the multi-layered MoS_2 grows and the flakes overlap.

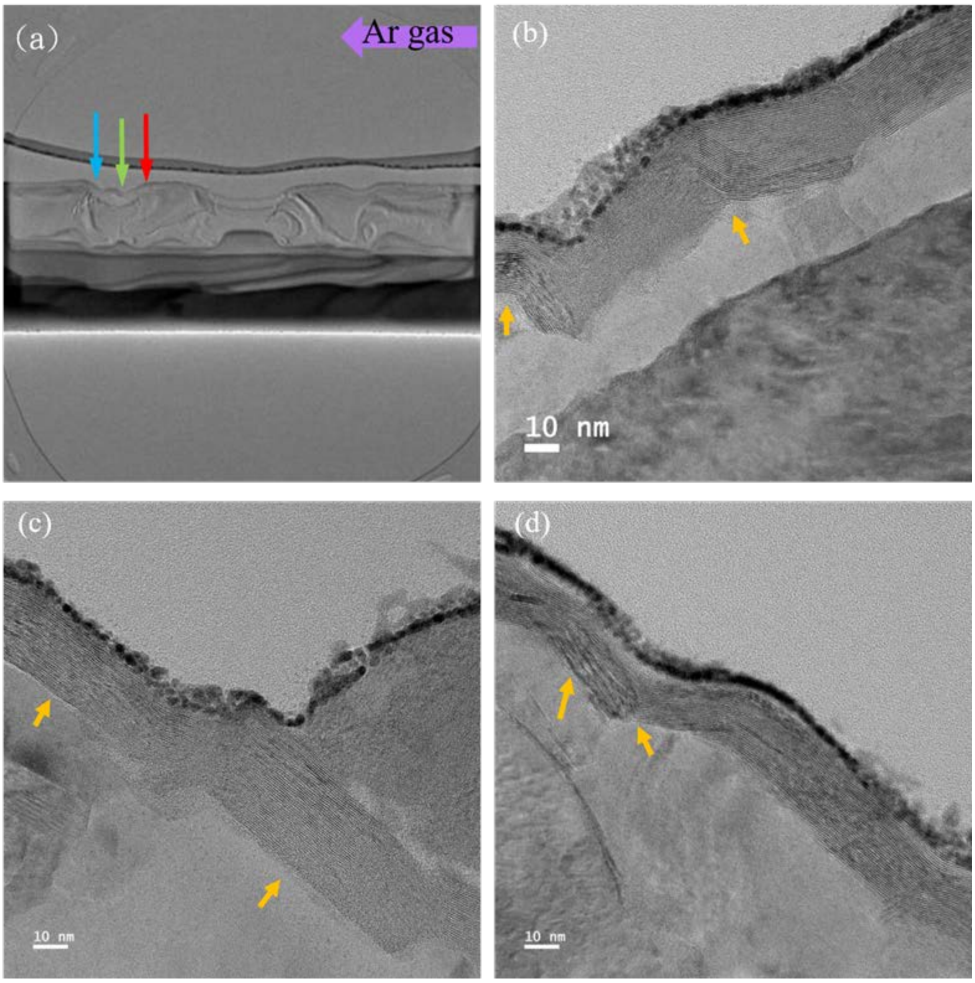

Figure S7. (a) is a schematic image of the cross section of sample#1, (b) indicates the HRTEM image at the red arrow in figure (a), (c) indicates the HRTEM image at the green arrow in figure (a), (d) indicates the HRTEM image at the blue arrow in figure (a).

Figure S8(a) is a schematic image of the cross-section of sample#1. The arrows of different colors will be used for HRTEM images. The purple arrow to the left represents the flow direction of argon. Figure S8(b) is the HRTEM image marked with the red arrow in Figure S8(a), and the yellow arrow is the multi-layer MoS_2 growth. Figure S8(c) is the HRTEM image marked by the blue arrow in Figure S8(a). The sampling position is outside. Although the yellow arrow marks the growth of multiple layers of MoS_2, the number of layers is significantly less. According to our observation, the overall number of layers of MoS_2 growth is getting thicker in terms of the arrow in the direction of the airflow. It can be clearly found that the number of layers along the airflow to the hole is about 7 to 10 layers. After the hole, the number of layers of MoS_2 deposition is 20 to 30 layers and the number of layers is reduced to 1 to 3 layers after the edge of the ring. The growth near the hole and the ring is more complicated than it contains many mixed areas.

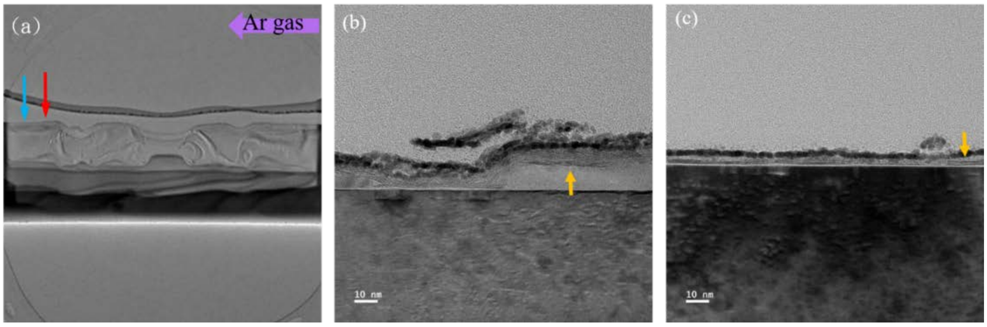

Figure S8. (a) is a schematic image of the cross section of sample#1, (b) indicates the HRTEM image at the red arrow in figure (a), (c) indicates the HRTEM image at the blue arrow in figure (a).

3.2. Composition analysis results after the growth of MoS_2

To analyse the growth of MoS_2, the use of energy dispersive spectrometer (EDS) is the most efficient method. By the impact of the electron beam, the X-ray of the elements is generated, and analyze based on the horizontal distribution of the elements in the imaged area drawn and the proportion of the generated data. Figure S9(a) is the TEM image of the cross-section of the substrate without MoS_2 periodic holes. The area circled by the green box is the result of the damage to the surface structure of the sapphire substrate during laser processing. Figure S9(b) is a TEM image of the cross-section of the substrate with periodic holes in the grown MoS_2. The red and yellow arrows on the two images represent the same part through observation, and the part in the green box is heated in the high-temperature furnace tube. Since the crystal structure has been destroyed with the CVD reaction, there will be part of the mixed area covered on the substrate. EDS is used to materialize the entire substrate cross-section of the grown MoS_2 periodic holes analyze.

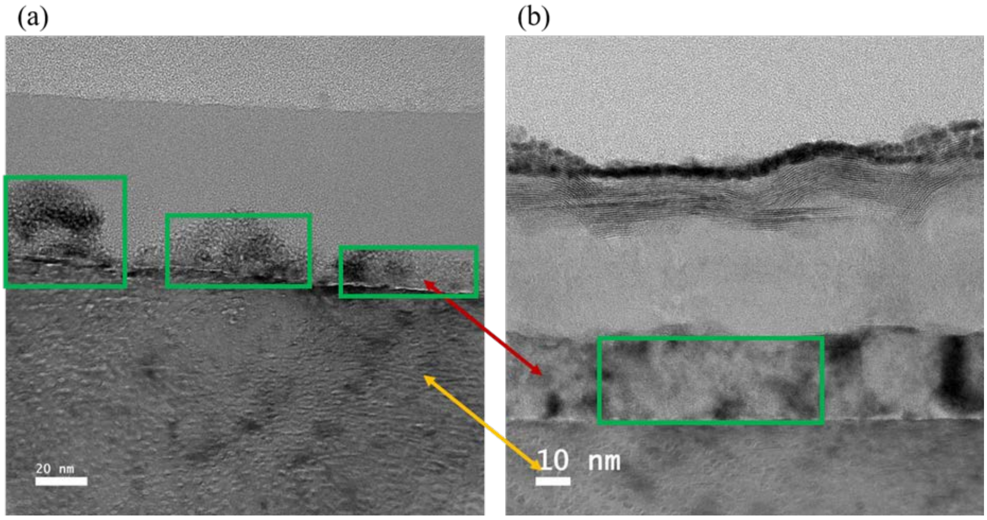

Figure S9. (a) The TEM image of the cross-section of the substrate without periodic MoS_2 holes, and (b) the TEM image of the cross-section of the substrate with MoS_2 periodic holes.

Figure S10(a) is the HRTEM image of the cross-section of the substrate with periodic holes in MoS_2 that has grown. Each color box is the part for EDS analysis. It is divided into five parts, as shown in the figure. Figure S10(b) is the EDS analysis diagram and data in the red box of Figure S10(a). The selected area is the surface layer. The highest ratio of Mo,

S, and O is observed from the graph. It is estimated that the main composition of the compound is MoS_2 and MoO_3. Figure S10(c) is the EDS analysis diagram and data in the orange box of Figure S10(a). The selected area is the MoS_2 multilayer growth distribution. We can infer from the data below, it can be determined that it is almost entirely composed of MoS_2. Figure S10(d) is the EDS analysis diagram and data in the yellow box of Figure S10(a). It is calculated from the percentage distribution of Mo, O and Al that it is possible that MoO_3 and Al_2O_3 form a mixture region. Figure S10(e) is the EDS analysis diagram and data of the green box in Figure S10(a). The selected area is at the junction of the mixed zone and another material. The composition is estimated to be composed of Al_2O_3, MoO_3 and MoS_2 based on the percentage of elements. Among them, MoS_2 was measured close to the sapphire substrate. The image is still not easy to see, and it is most likely to be a single layer or low-layer growth. Figure S10(f) is the EDS analysis diagram and data in the blue box of Figure S10(a). The percentage of the elements can be clearly seen to be composed of Al_2O_3.

As shown in Figure S10(e), there is growth of MoS_2 near the sapphire substrate. EDS analysis was also conducted at the hole close to the substrate, as shown in Figure S11.

(a)

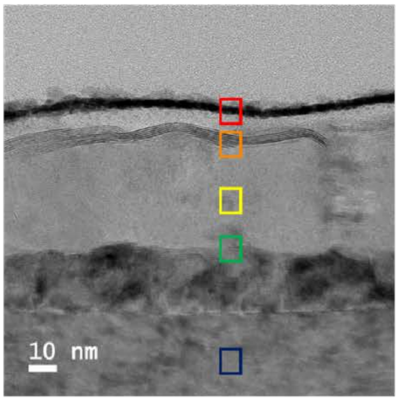

(b)

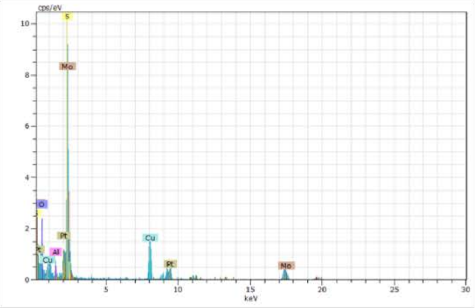

Spectrum: 1

Element	Series	Net	unn. C [wt.%]	norm. C [wt.%]	Atom. C [at.%]	Error (3 Sigma) [wt.%]
Oxygen	K-series	313	8.01	8.01	23.20	1.61
Aluminium	K-series	73	1.58	1.58	2.71	0.65
Sulfur	K-series	1487	31.61	31.61	45.68	3.89
Molybdenum	K-series	312	58.81	58.81	28.41	12.19
		Total:	100.00	100.00	100.00	

(c)

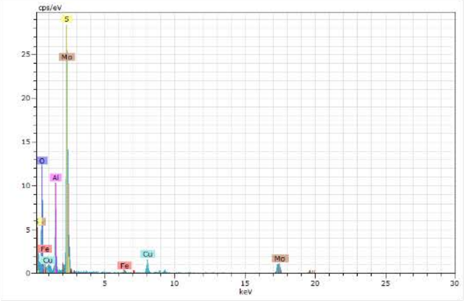

Spectrum: 2

Element	Series	Net	unn. C [wt.%]	norm. C [wt.%]	Atom. C [at.%]	Error (3 Sigma) [wt.%]
Oxygen	K-series	1082	14.51	14.51	32.97	1.93
Sulfur	K-series	2709	30.05	30.05	34.07	3.32
Molybdenum	K-series	430	42.31	42.31	16.03	7.76
Aluminium	K-series	1083	12.04	12.04	16.23	1.62
Iron	K-series	73	1.08	1.08	0.71	0.49
		Total:	100.00	100.00	100.00	

(d)

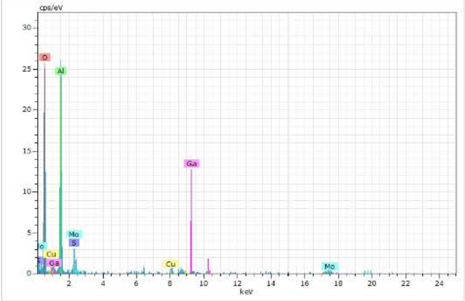

Spectrum: 3

Element	Series	Net	unn. C [wt.%]	norm. C [wt.%]	Atom. C [at.%]	Error (3 Sigma) [wt.%]
Oxygen	K-series	429	43.61	43.61	61.16	7.52
Aluminium	K-series	502	42.49	42.49	35.33	6.96
Sulfur	K-series	6	0.54	0.54	0.38	0.74
Molybdenum	K-series	17	13.37	13.37	3.13	10.50
		Total:	100.00	100.00	100.00	

(e)

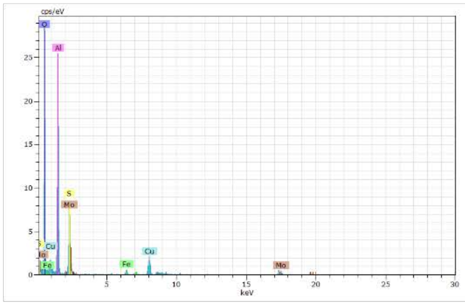

Spectrum: 4

Element	Series	Net	unn. C [wt.%]	norm. C [wt.%]	Atom. C [at.%]	Error (3 Sigma) [wt.%]
Oxygen	K-series	1612	39.87	39.87	59.17	4.74
Aluminium	K-series	1662	34.10	34.10	30.01	4.05
Molybdenum	K-series	89	16.22	16.22	4.01	5.88
Sulfur	K-series	408	8.36	8.36	6.19	1.56
Iron	K-series	53	1.45	1.45	0.61	0.72
		Total:	100.00	100.00	100.00	

(f)

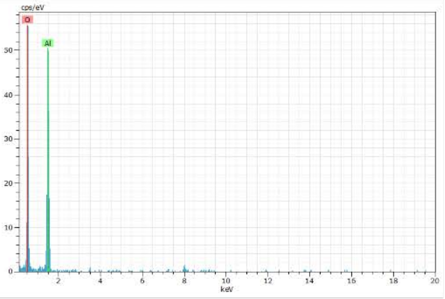

Spectrum: 5

Element	Series	Net	unn. C [wt.%]	norm. C [wt.%]	Atom. C [at.%]	Error (3 Sigma) [wt.%]
Oxygen	K-series	635	52.18	52.18	64.79	7.87
Aluminium	K-series	701	47.82	47.82	35.21	7.02
		Total:	100.00	100.00	100.00	

Figure S10. EDS analysis diagram and data in the green box in Figure (a), and (f) is the EDS analysis and data in the blue box in Figure (a).

Figure S11(a) is the TEM image of the cross-section of the substrate with periodic holes after growing MoS_2. The arrow point is the hole for EDS analysis. Figure S11(b)

is the HRTEM image of the part indicated by the arrow in Figure S11(a). Figure S11(c) is the EDS analysis diagram and data of the blue box in Figure S11(b). The composition of the compound is analyzed by element ratio, and it is composed of Al_2O_3, MoO_3 and MoS_2.

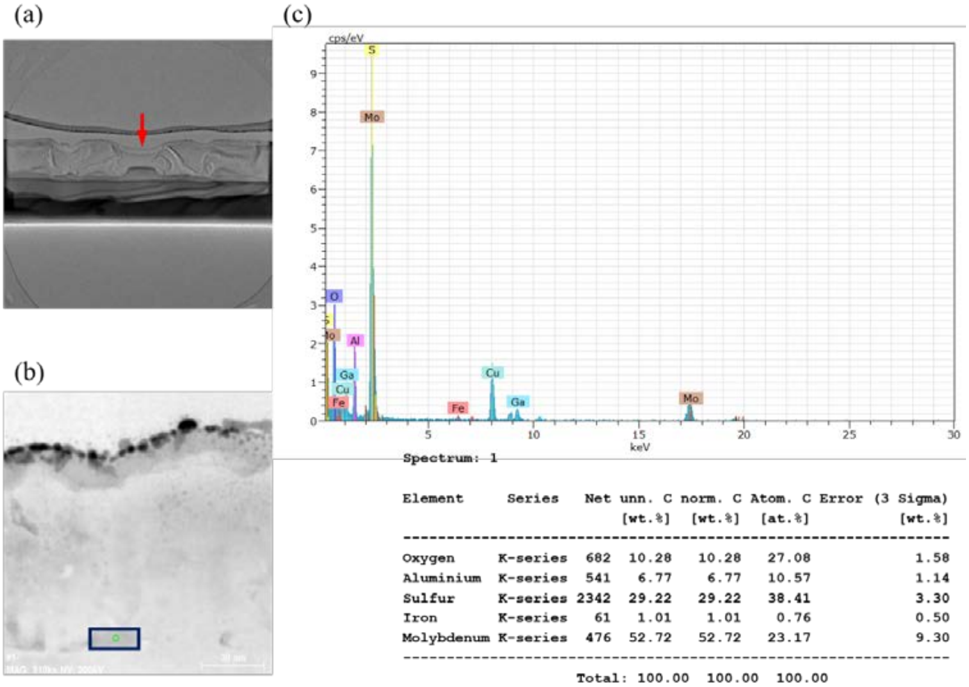

Element	Series	Net	unn. C [wt.%]	norm. C [wt.%]	Atom. C [at.%]	Error (3 Sigma) [wt.%]
Oxygen	K-series	682	10.28	10.28	27.08	1.58
Aluminium	K-series	541	6.77	6.77	10.57	1.14
Sulfur	K-series	2342	29.22	29.22	38.41	3.30
Iron	K-series	61	1.01	1.01	0.76	0.50
Molybdenum	K-series	476	52.72	52.72	23.17	9.30
		Total:	100.00	100.00	100.00	

Figure S11. TEM image of the cross-section of the substrate with periodic holes after growing MoS_2. The arrow is the hole for EDS analysis, (b) is the HRTEM image of the part indicated by the arrow in the figure (a), and (c) is the EDS analysis diagram and data in the blue box in Figure (b).

According to the above EDS analysis we can conclude that when the vulcanization rate is faster than the growth rate of MoS_2, the dominance S is seen, which is the growth mechanism of single-layer MoS_2; when the vulcanization rate is slower than the growth rate of MoS_2, the dominant of Mo-oxysulfide can be seen, which is the growth mechanism of muti-layered MoS_2. From our experimental setup, 1 g of sulfur is piled into a cone, and 400 sccm of Ar gas is blown to the crucible where the sapphire substrate and MoO_3 are set up. In the beginning, the gas drives the sulfur faster, and a small amount of single-layer MoS_2 grows. Later, limited by the height the speed of driving the sulfur slows down, and the deposition of MoO_3 is mixed with Al_2O_3 to form a mixed zone. Among them, the mixing of Al_2O_3 is because the structure of the surface sapphire substrate is destroyed by laser processing to make the substrate hole. It forms a mixture with other compounds when heated, and it grows to the later stage because MoO_3 is nearly exhausted. With S as the leading factor, MoS_2 grows close to the surface.

Author Contributions: Conceptualization, V.E.E, Y.-M.T, H.-C.W. and S.Y.H.; methodology, A.M., H.-C.W., S.Y.H and Y.-M.T; software, S.Y.H., V.E.E, H.-C.W. and A.M.; validation, Y.-M.T, V.E.E, S.Y.H. and H.-C.W.; formal analysis, Y.-M.T, V.E.E and H.-C.W.; investigation, Y.-M.T, V.E.E and S.Y.H.; resources, Y.-M.T, V.E.E and H.-C.W.; data cura-tion, A.M, H.-C.W., S.Y.H and S.Y.H.; writing—Original draft preparation, A.M, ; writing—Review and editing, A.M, and H.-C.W.; supervision, H.-C.W.; project administration, H.-C.W. All authors have read and agreed to the published version of the manuscript.

Conflicts of Interest: The author declare no conflict of interest.

Funding: This research was supported by the Ministry of Science and Technology, The Republic of China under the grants MOST 105-2923-E-194-003 MY3, 108-2823-8-194-002, 109-2622-8-194-001-TE1, and 109-2622-8-194-007. This work was financially/partially supported by the Advanced Institute of Manufacturing with High-tech Innovations (AIM-HI) and the Center for Innovative Research on Aging Society (CIRAS) from The Featured Areas Research Center Program within the framework of the Higher Education Sprout Project by the Ministry of Education (MOE) in Taiwan.
